# A nationwide serological survey for *Dirofilaria immitis* in companion cats in the United States of America: 3.5% antibody and 0.3% antigen positivity

**DOI:** 10.1186/s13071-023-05829-7

**Published:** 2023-08-24

**Authors:** Daniel Felipe Barrantes Murillo, Lindsay Starkey, Theresa Wood, Rachel Smith, Byron Blagburn, Joy Bowles, Hill Allen, Caroline Lewis, Yue Shu, Chengming Wang

**Affiliations:** 1 Department of Pathobiology, Auburn University College of Veterinary Medicine, Auburn, AL USA; 2 College of Sciences and Mathematics, Auburn University, Auburn, AL USA

**Keywords:** *Dirofilaria immitis*, Companion cats, USA, Antigen testing, Antibody detection, Acid treatment

## Abstract

**Background:**

Feline heartworm disease (HWD) is a complex and often misdiagnosed disease in cats, caused by the filarial nematode *Dirofilaria immitis*. Despite its significant impact, studies reporting the prevalence of *D. immitis* in apparently healthy pet cats in the USA are lacking.

**Methods:**

To investigate feline heartworm seroprevalence in apparently healthy pet cats in the USA, serum samples (*n* = 2165) collected from cats across 47 states and Washington District of Columbia were analyzed for *D. immitis* antibody (Heska Corp.) and antigen (DiroCHEK®; Zoetis Inc.) with and without acid treatment of the samples.

**Results:**

Antibodies to *D. immitis* antibodies were identified in 3.5% (76/2165) of cats from 26 states, with a significantly higher prevalence in cats from the westernmost US states (West region; 5.4%, 23/429) compared to those from the South (3.8%, 32/847), Midwest (2.7%, 9/338) and Northeast regions (2.2%, 12/551) (*P* < 0.04). Antigen from *D. immitis* was detected in 0.3% (6/2165) of cats, which was significantly lower than the antibody detection (*P* < 10^–4^), and no samples were positive for both antibody and antigen.

**Conclusions:**

This is the largest antibody-based, nationwide serosurvey of feline heartworm in an apparently healthy cat population, and the results suggest that cats in the USA have a high risk of exposure to *D. immitis*-infected mosquitoes. The high nationwide prevalence (3.5%) indicates that the true prevalence of cats infected with *D. immitis* in the USA may be significantly underestimated. Our findings emphasize the need for increased awareness and routine testing of cats for heartworm infection, especially in non-endemic areas of the USA. Clinicians should consider appropriate use of broad-spectrum veterinary-approved parasiticides and lifestyle management in feline patients to reduce the risk of infection. Future studies should focus on evaluating the *D. immitis* infection status in healthy cats and developing better diagnostic assays to detect this complex infection.

**Graphical Abstract:**

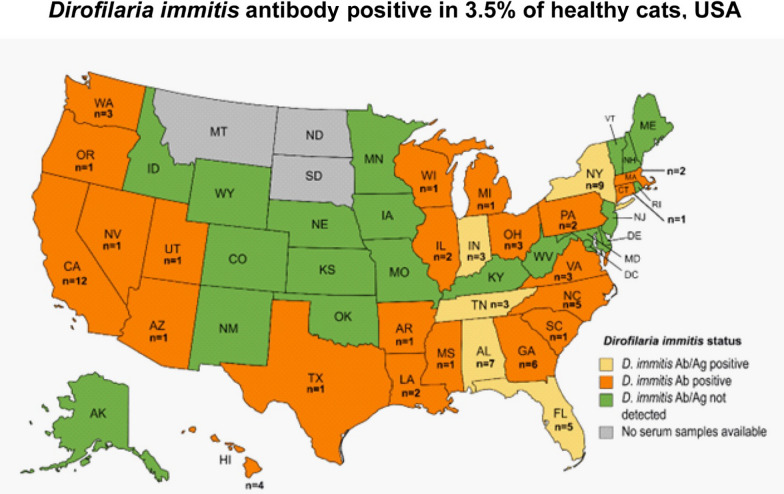

**Supplementary Information:**

The online version contains supplementary material available at 10.1186/s13071-023-05829-7.

## Background

Feline heartworm disease (HWD) is caused by infection with the filarial nematode, *Dirofilaria immitis,* after transmission of the third-stage larvae by a competent mosquito vector [1, 2]. The infective larvae enter the skin through the bite wound of the mosquito, molt to fourth-stage larvae and then to immature adults before they migrate to the arteries and arterioles of the lungs, 70–90 days post-infection [1]. Aberrant migration of fourth-stage larvae has been reported in cats' body cavities and central nervous system [3]. Unlike dogs, cats are not considered the final host and do not serve as a natural reservoir of this infectious agent [1, 2].

*Dirofilaria immitis* infection in cats is responsible for two distinct clinicopathological syndromes, heartworm-associated respiratory disease (HARD) and feline adult HWD [1, 4, 5]. HARD is caused by the death of immature worms that leads to vascular, interstitial, bronchial and bronchiolar histological lesions in the lung [1, 4]. Clinical signs of cats with HARD include lethargy, coughing, tachypnea, vomiting and respiratory distress [2]. Feline HWD is a consequence of ineffective clearance of heartworms by the cat’s immune system [1, 6]. Usually, only one to two adult worms are present in cats with HWD, and microfilaremia is rare and transitory [7]. Adult parasites can live 2–4 years in the feline host [7]. Severe inflammation, thromboembolism and death can occur in feline patients after adult worms die. Other clinical signs are respiratory distress, pulmonary hemorrhage, syncope, ataxia and seizures [8].

Cats can be infected by *D. immitis* at any age, and the infection is not exclusive to outdoor cats [2]. In fact, in a previous study investigating risk factors among a cohort of infected cats, 27% of the *D. immitis*-infected cats reportedly lived indoors [2]. The prevalence of HWD in cats is estimated to be 5–10% of the reported canine HWD prevalence from the same area and can be as high as 20% in some locations [2, 8, 9]. The Companion Animal Parasite Council (CAPC) reported an overall prevalence of 0.61% of positive cases by the antibody test and of 0.57% of positive cases by the antigen test in the continental USA in 2022 [10].

The diagnosis of feline heartworm (HW) infection requires a multimodal approach that includes the correlation of clinical signs, thoracic radiographs, echocardiography, antigen and antibody serology [1, 2]. Serological tests must be interpreted carefully with an appropriate awareness of each test's limitations [4, 8]. Third-stage larvae successfully transmitted by the mosquito to a cat should yield a positive antibody test; however, the test itself indicates a history of infection and does not delineate between previous or current infection status at the time of the test [4, 11]. Although the duration of the antibody response is variable among cats, serological detection of antibodies can be reached as early as 8 weeks post-infection and be consistently positive for at least 5 months [2, 8, 11].

Diagnosis of HARD in cats can only be supported with consistent clinical signs in addition to a positive antibody test [12]. For the diagnosis of adult worms, the antigen test is preferred because it detects the antigen secreted by female adult worms [13]. A negative antigen test does not necessarily rule out diagnosis of adult female heartworms in a cat. While a negative test can truly reflect the absence of adult worms, other situations in which a false negative may occur include if the infection consists of only immature stages, the worm burden is too low, the worm population has few to no mature females or the antigen is bound in immune complexes and rendered not detectable by the assay [13–15]. Furthermore, less than 20% of cats with adult HW infections have detectable microfilaremia, which is only detectable 1–2 months after maturation of adult worms, which is about 7–8 months post-infection by the third-stage larvae [8, 11, 16].

In the present study, we aimed to provide additional information regarding the epidemiological distribution of *D. immitis* in a presumably healthy population of owned cats through the well-standardized commercial diagnostics for antibody and antigen detection. Additionally, we used an acid-based immune complex dissociation (ICD) protocol on each sample [17] before antigen testing. To the best of our knowledge, acid treatment before antigen testing has not been used on feline samples, and we elected for this protocol as an alternative to heat treatment due to the limited volume of available serum from each cat. The prevalence of HW exposure in cats is not well understood, and previous studies have reported widely varying prevalence rates, ranging from < 1% to > 20%. These discrepancies may be due to differences in study design, sample size and geographical location. Therefore, there is a need for more accurate and comprehensive data on the prevalence of HW exposure in cats in the USA. Such data can help inform strategies for prevention, diagnosis and treatment of this important disease in feline patients.

## Methods

### Serum samples from cats

In this study, serum samples from apparently healthy companion cats (*n* = 2165) were used. These samples were submitted to Auburn University College of Veterinary Medicine between January 2017 and May 2022 for rabies antibody titer determination as a requirement for international or domestic travel. The samples were submitted from 47 states of the continental USA and the District of Columbia. However, no samples were received from the states of Montana, South Dakota and North Dakota (Table 1). The samples were sorted by geographical location into four regions based on the geographic region definitions of the U.S. Centers for Disease Control and Prevention (CDC; https://www.cdc.gov/nchs/hus/sources-definitions/geographic-region.htm). Information on the sex, age, breed and geographic location of cats was retrieved for each case.

**Table 1:** Seroprevalence for *Dirofilaria immitis* from the companion cats included in this study

State^a^	Total samples (n)	Number and percentage of antibody-positive samples^b^ %	Number and percentage of antigen-positive samples^b^%
Alabama	172	6 (3.4)	1 (0.6)
Arizona	20	1 (5.0)	0 (0.0)
Arkansas	25	1 (4.0)	0 (0.0)
California	191	12 (6.2)	0 (0.0)
Connecticut	16	1 (6.2)	0 (0.0)
Florida	195	4 (2.0)	1 (0.5)
Georgia	105	6 (5.7)	0 (0.0)
Hawaii	43	4 (9.3)	0 (0.0)
Illinois	54	2 (3.7)	0 (0.0)
Indiana	28	2 (7.1)	1 (3.6)
Louisiana	12	2 (16.6)	0 (0.0)
Massachusetts	140	2 (1.4)	0 (0.0)
Michigan	59	1 (1.7)	0 (0.0)
Mississippi	8	1 (12.5)	0 (0.0)
North Carolina	47	5 (10.6)	0 (0.0)
Nevada	23	1 (4.3)	0 (0.0)
New York	189	7 (3.7)	2 (1.1)
Ohio	88	3 (3.4)	0 (0.0)
Oregon	8	1 (12.5)	0 (0.0)
Pennsylvania	138	2 (1.4)	0 (0.0)
South Carolina	21	1 (4.7)	0 (0.0)
Tennessee	39	2 (5.1)	1 (2.6)
Texas	107	1 (0.9	0 (0.0)
Utah	3	1 (33.3)	0 (0.0)
Virginia	77	3 (3.9)	0 (0.0)
Washington	82	3 (3.6)	0 (0.0)
Wisconsin	25	1 (4.0)	0 (0.0)
Total	2,165	76 (3.5)	6 (0.3)

^a^*Dirofilaria immitis* antibody and antigen testing results were negative for an overall total of 248 cats from Alabama (*n* = 2), Colorado (22), Washington DC (8), Delaware (3), Iowa (16), Idaho (27), Kansas (5), Kentucky (10), Maryland (13), Maine (8), Minnesota (31), Missouri (22), Nebraska (10), New Hampshire (11), New Jersey (43), New Mexico (3), Oklahoma (2), Rhode Island (2), Vermont (4), West Virginia (3) and Wyoming (3)

^b^Percentage is given in parentheses

Each serum sample had been previously heated at 56 °C for 30 min for fluorescent antibody virus neutralization (FAVN) test and rapid fluorescent foci inhibition test (RFFIT) and stored at − 20 °C, prior to being thawed at room temperature for this study. After thawing, three aliquots for each sample were taken as follows: 50 µl for antibody testing, 50 µl for antigen testing and 100 µl for acid treatment followed by antigen testing.

### Antibody test detection

To detect *D. immitis* antibodies, a 50-μl aliquot of each cat sample was submitted to Heska Corp. (Loveland, CO, USA) for testing. The company’s Feline Heartworm Antibody test, based on an enzyme-linked immunosorbent assay (ELISA), was used, performed at the Veterinary Diagnostic Laboratory of Heska Corp. The test has been validated for its sensitivity and specificity, and a single highly purified proprietary antigen was used in the ELISA. Any values ≥ 5 antibody units per milliliter (AbU/ml) were considered to be positive.

### Antigen testing with and without acid treatment

For HW antigen detection, a non-acid-treated aliquot and a post-acid-treated aliquot of each sample were evaluated using a well-based ELISA (DiroChek® Heartworm Antigen Test Kit; Zoetis, Florham Park, NJ, USA) according to the manufacturer’s instructions. Acid treatment was used as the method for ICD as previously described [17]. Briefly, 100 μl of serum and 100 μl of 7.5% (*w*/*v*) trichloroacetic acid (TCA) were mixed in a 1.5-ml microcentrifuge tube, followed by incubation at room temperature for 20 min, centrifugation and recovery of the supernatant. A 150-μl aliquot of the centrifuged sample was then mixed with 30 μl of 1 M Trizma buffer to return the sample to a neutral pH. The presence of HW antigen was visually determined by a color change on the DiroCHEK®, as indicated in the manufacturer’s instructions.

### Statistical analysis

The statistical analyses were conducted using the IBM® SPSS® Statistics software package (IBM Corp., Armonk, NY, USA). A Chi-square (*χ*^2^) test was performed to compare the prevalence of *D. immitis* antibody and antigen testing between cats from different geographical regions, genders, age groups and years of submission. A difference at *P* ≤ 0.05 was considered to be statistically significant.

## Results

**Fig. 1:**
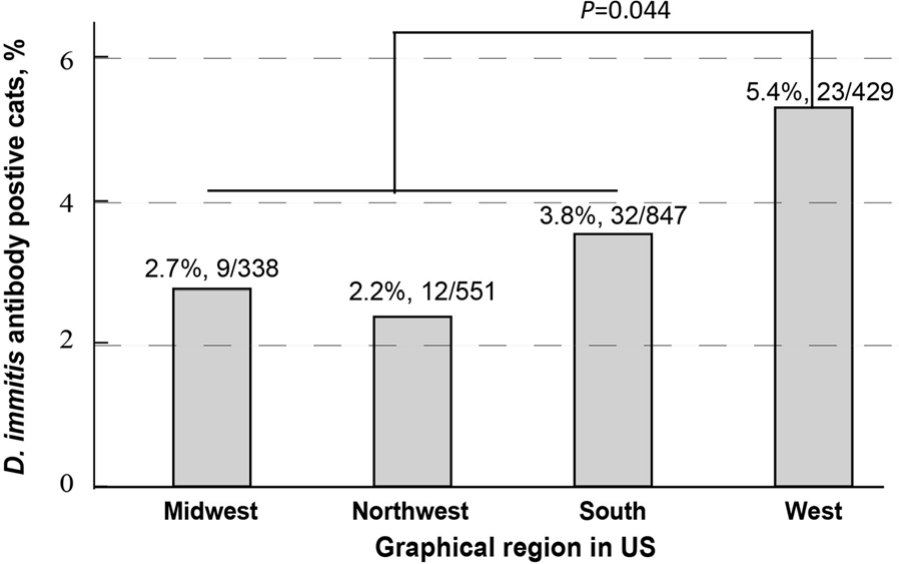
Geographical distribution by region of *Dirofilaria immitis* antibody- and antigen-positive healthy companion cats. The prevalence in cats from the West region of the USA (5.4%, 23/429) was significantly higher than that in cats from the South (3.8%, 32/847), Midwest (2.7%, 9/338) and Northeast (2.2%, 12/551) regions (*χ*^2^ test, *P* = 0.044). A difference at *P* ≤ 0.05 was considered to be statistically significant. The definition of the different regions can be found at: https://www.cdc.gov/nchs/hus/sources-definitions/geographic-region.htm

In this study, Heska's Feline Heartworm Antibody test identified an overall *D. immitis* antibody prevalence of 3.5% (76/2165) in cats from 26 of 47 states (55.3%) (Table 1; Fig. 1; Additional file 1: Table S1). The prevalence of *D. immitis* antibody in cats from the West region of the USA (5.4%, 23/429) was significantly higher than that in cats from the South (3.8%, 32/847), Midwest (2.7%, 9/338) and Northeast (2.2%, 12/551) regions (*χ*^2^ test, df = 1, *P* = 0.044) (Fig. 1).

The Domestic shorthair was the most represented breed among the antibody-positive samples (*n* = 44), followed by the Domestic longhair (7) and Sphynx (4) (Additional file 1: Table S1). The Ragdoll and British shorthair breeds were represented by three individuals, whereas the Domestic medium hair, Persian, Scottish Fold and Siamese breeds were represented by two individuals. Only one cat of each of the following breeds was positive: British longhair, Calico, Siamese Mix and Tabby. The breed remained unknown for three positive samples (Additional file 1: Table S1). The mean age of the antibody-positive cats was 5.3 years (± 4.4 standard deviation [SD]) with an age range from 6 months to 19 years. The mean age for antibody-negative cats was 4.3 years (± 3.6 SD) with an age range from 1 month to 23 years.

**Fig. 2:**
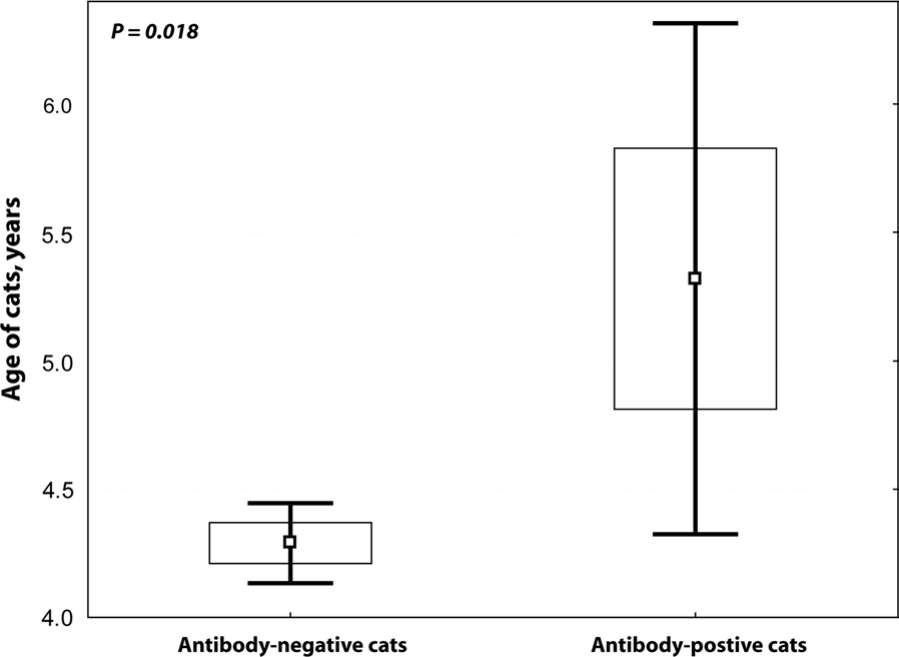
Cats positive for heartworm antibody were significantly older than the heartworm antibody-negative cats (mean ± standard deviation: 5.32 ± 4.37 vs 4.29 ± 3.63; *χ*^2^ test, *P* = 0.018). In addition, *Dirofilaria immitis* antibody-positivity was found in 31 of 1049 female cats and 46/1114 male cats (*χ*^2^ test, *P* = 0.074) (data not shown)

The distribution of the cases per year was 3.8% (13/343) in 2022, 3.9% (35/888) in 2021, 2.4% (14/574) in 2020, 4.0% (10/252) in 2019, 4.5% (3/67) in 2018 and 2.6% (1/38) in 2017 (Additional file 1: Table S1). The cats which tested positive for HW antibody were significantly older than those which were HW antibody negative (mean ± SD: 5.32 ± 4.37 vs. 4.29 ± 3.63; *χ*^2^ test, *P* = 0.018). The difference in the prevalence of feline HW was not statistically significant by sex (female: 3.0%, 31/1039; male: 4.0%, 45/1123; *P* = 0.07) (Fig. 2). Additionally, *D. immitis* antibody-positivity was found in 31 of 1049 female cats and 46 of 1114 male cats (*χ*^2^ test, *P* = 0.074).

**Fig. 3:**
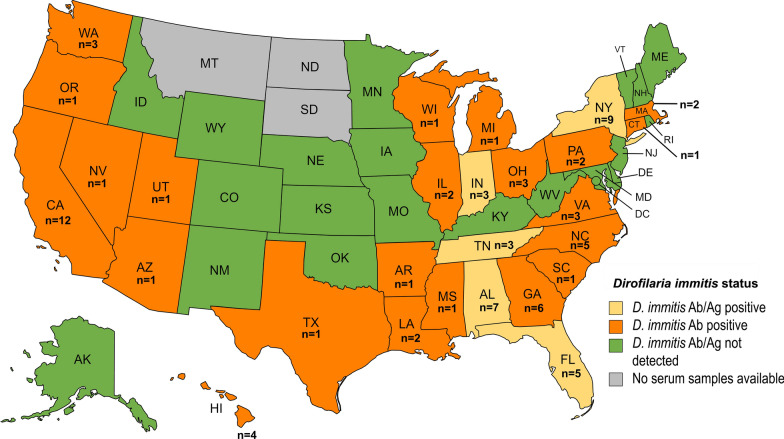
Geographical distribution by state of *Dirofilaria immitis* antibody and antigen positive healthy companion cats. Heska’s Feline Heartworm Antibody test identified an overall prevalence of 3.5% (76/2165) antibody positivity in 26 of the 47 states and Washington District of Columbia (55.3%) included in this study. The overall prevalence of *D. immitis* antigen detection with and without acid treatment was 0.3% (6/2165)

The DiroChek® Heartworm Antigen Test Kit detected antigen in 0.2% (4/2165) of the samples, with positive samples originating from Alabama, New York and Tennessee (Fig. 3). After acid treatment, antigen was detected in 0.1% (2/2165) of the samples, and these were from Indiana and Florida. Thus four positive samples were detected before acid treatment, and two were detected after acid treatment. None of the samples that tested positive before acid treatment remained positive after acid treatment. The overall prevalence of antigen detection with and without acid treatment was 0.3% (6/2165), which is significantly lower than the antibody positivity (*P* < 10^–4^). Interestingly, no samples tested in this study were found to be both antigen and antibody positive. The antigen-positive samples were from 2020 (1/6) and 2021 (5/6), of which five were from female cats and one from a male cat.

## Discussion

Nationwide serosurveys on feline HW infection in the USA are not commonly conducted, and regional studies in endemic states are more frequent. In the USA, *D. immitis* infection in cats has been reported in 29 states [18]. For example, the CAPC reported an antibody seroprevalence of HW ranging from 0.61 to 1.19% from 2017 to 2022 [10]. Traditionally, the rate of positive samples from cats is about 10% of the canine rate in the same location [7, 19]. However, a retrospective study indicated that the feline positivity rate could be as high as 60% of the canine positivity rate in the same area [20]. The significant difference in prevalence between dogs and cats may be partially explained by differences in their lifestyles and in the parasite life-cycle in dog and cats, as well as by diagnostic assay performance and routine screening recommendations. Some authors argue that veterinary practitioners usually only test cats when they are suspected of being infected with HW, whereas healthy dogs are recommended to be screened annually [20].

This study found that the prevalence of *D. immitis* antibody seropositivity among the surveyed cats was 3.5%, which differs from the previously reported *D. immitis* antibody-positive prevalence that varies from 1.1% to 8% in one study [20], and a high antibody prevalence ranging from 11.8% to 14% [21, 22]. Previous prevalence surveys of *D. immitis* infection have been conducted on distinct animal populations; including animals from endemic areas, pet cats with clinical signs or free-roaming cats [13, 20, 23]. In contrast, the cats included in this survey were assumed to be healthy, and the samples for the cats included in this study were not believed to have been submitted due to suspected *D. immitis* infection. The finding that 3.5% of these healthy, owned cats surveyed showed antibody positivity indicates that these cats are exposed to HW infection more frequently than expected or previously reported.

HW testing in cats may be becoming a more common practice. The IDEXX Laboratories Reference Laboratory Network performed a nationwide retrospective study of the prevalence of HW in cats by retrieving the results from five diagnostic tests, including feline HW antigen and feline HW antibody, from patients with clinical suspicion of HW infection [20]. The database included all of the results between January 2000 and April 2007. The authors examined the zip code of each sample and demonstrated that feline HW antigen and antibody testing is widely distributed in the USA. They also found that the number of feline samples submitted for antigen and antibody HW testing increased every year of the study period, with an annual growth rate of 21% and 32%, respectively. The average prevalence of HW infection in cats ranged from 0.5% to 1.4% (average 0.9%) determined by the antigen test, and from 1.1% to 8% (average 4.2%) determined by the antibody test [20]. Another nationwide serosurvey included samples from 34,975 cats (26,441 from veterinary clinics and 8125 from shelters) from all 50 U.S. states in 2010 [24]. Antigen of *D. immitis* was detected in 0.4% of all cats using a commercial ELISA kit (SNAP feline triple test; IDEXX Laboratories Inc, Westbrook, ME, USA) [24]. The seroprevalence was 0.3% in healthy cats and 1.0% in cats with respiratory signs [24]. The reported antigen prevalence in these two nationwide surveys (0.9% in 2000–2006 and 0.4% in 2010) is similar to our reported rate (0.3%), bearing in mind that the previous studies’ sample sets included a mix of non-healthy and healthy cats.

Serological tests are considered to be the most useful for diagnosing HW infection in cats [25] although the test results must be interpreted with caution. In our study, there was a notable difference between the overall antigen-positive rate (0.3%) and the antibody-positive rate (3.5%). This can be explained by the limitations of the antigen test and the unique features of HW infection in cats. Even if the antigen is not detected, the nature of feline HW infection makes a negative antigen test result unreliable for conclusively ruling out HW infection from diagnosis; rather, the correct interpretation of a negative antigen test is reported as “no antigen detected.” A false-negative result is attributed to the lack of antigen detection in cats due to the low burden of parasites, male-only infections, the lack of development of antigen-producing adult worms and/or immune-complex formation rendering the antigen undetectable [26, 27]. Alternatively, false-positive results can be obtained when the remaining circulating antigen is present in the blood circulation weeks after the clearance of adult worms, resulting from the death and decomposition of male or female filarial worms or by non-*D. immitis* substances present in the sample that erroneously elicit a positive result on the test [2].

In this study, an acid-based ICD protocol was used to increase the sensitivity for detecting antigen. Heat- or acid-based ICD protocols are commonly used to improve the detection of antigen in feline samples [28, 29]. While heat treatment is recommended for samples obtained from cats from endemic areas or those without prophylactic treatment, few studies have looked at its efficacy in cats. Some studies have reported an increase in antigen detection after heat treatment, while others have contradicted these findings, reporting no change in antigen detection rates [30, 31]. In contrast, acid treatment is not widely used in cats for HW detection. To our knowledge, our study is the first to apply an acid treatment protocol in a large subset of feline samples (*n* = 2165). We preferred this ICD protocol to heat treatment due to the limited volume of serum available from each cat. In our study, all samples that initially tested positive for HW antigen became negative following acid treatment, while only two additional samples initially negative converted to positive. Since antigen levels in cats may be lower due to low worm burdens, the conversion to negative antigenicity following acid treatment could be due to sample dilution. It is also possible that the ICD treatment may lead to changes in the structural and antigenic properties of proteins. However, due to the small number of samples testing positive either before or after acid treatment and the unknown infection status of the cats, we cannot conclude if the acid treatment is beneficial for feline HW diagnosis. Further controlled experiments using animals of known infection status should be conducted to compare acid and heat ICD methods versus no ICD treatment in feline samples.

There was no overlap between antigen-positive and antibody-positive samples in this study. It is important to note that a positive antibody test only indicates exposure and does not necessarily mean that the life-cycle of the parasite has been completed or that adult parasites are present in the heart or lungs [2]. Feline patients may have elevated levels of antibodies for weeks or months after mounting an immunological response and parasite clearance [2]. A negative antibody test indicates that the cat is not infected, was infected < 50–60 days ago or has antibodies that fall below the detectable limits of the diagnostic assay [2]. While we cannot determine the specific stage of the infection, it is unlikely that these cats had an active infection since the samples were submitted for pre-travel rabies titer determination and, therefore, the cats were unlikely to be exhibiting any clinical signs. However, some cats affected with feline cardiopulmonary dirofilariasis are asymptomatic [16, 32]. It is also important to keep in mind that antibody testing detects a different aspect of the infection and can overestimate the number of actively infected animals [13].

Studies on infected cats have shown that several risk factors are associated with HW infection, identified using various diagnostic approaches such as post-mortem examination, antibody and antigen testing and the modified Knott’s test. Male cats, domestic shorthair cats, cats with underlying health conditions and cats with outdoor access are considered to be at increased risk, while indoor cats are considered to be partially protected [24, 25]. In the present study, breed and sex were not found to be statistically significant risk factors, unlike age, where older cats had a higher tendency to be antibody positive. Data on outdoor access were not available for this study.

The breed distribution of the antibody-positive samples in this study was consistent with that reported in previous studies, with the Domestic shorthair being the most commonly affected breed [25]. The mean age of antibody-positive cats was higher than that of antibody-negative cats, which is also consistent with the results of previous studies that have reported an association between feline HW disease and increasing age [25].

Another important predisposing factor for feline HW disease is the geographical location. Seroprevalence is considered to be higher in the Southern region of the USA, followed by the Midwest; the regions with the lowest rates are reported to be the Northeast and West [24]. In our study, a higher prevalence of antibody-positive *D. immitis* samples occurred in the West region of the USA, which contrasts with the previously reported high incidence in the Southern region by antibody testing [24]. In theory, feline *D. immitis* infection prevalence should be high in the geographical locations in which canine dirofilariasis is endemic or hyperendemic [28, 29]. Interestingly, no antigen-positive samples were identified from the West region of the USA in the present study. Human activities, such as irrigation and tree plantations, have been directly responsible for the expansion of the territory for several mosquito species, such as *Aedes sierrensis* which is known to be a principal vector of HW in the West region of the USA [33]. Moreover, some researchers believe that the increased prevalence of HW in the USA is due to the traveling or relocation of microfilaremic domestic or wild dogs to non-endemic regions creating a local reservoir host, thus promoting autochthonous transmission [27, 34]. Climate change induced by human activities can create microenvironments for the vectors to subsist and expand their geographical ranges could also be a contributing factor [27, 34].

One limitation of this study is sampling bias, with sample distribution being uneven among the states. The state of California was overrepresented with 191 samples, whereas states from the Southeast, like Mississippi, Louisiana and South Carolina, were represented with < 50 samples each. Another limitation is the inability to obtain travel history as only the zip code of veterinarian submitting for the rabies titer was known and used for mapping. This could impact the geographic trends noted in this study. Our findings suggest that cats in the West region are more likely to be exposed to *D. immitis* infection in comparison with those in the South region. It is important to note that the limitations of this study may have impacted the results, and further research is needed to fully understand the prevalence and distribution of feline HW infection in the USA. Nonetheless, the findings suggest that there may be regional differences in the prevalence of feline HW infection, and that geographical location, as well as other risk factors such as age and health status, should be taken into consideration when testing cats for HW infection. Veterinarians should continue to educate cat owners about the importance of HW prevention and testing and work to develop tailored prevention strategies based on the individual risk factors of each patient.

## Conclusions

In conclusion, this study represents the largest nationwide survey of an apparently healthy cat population, incorporating both antibody and antigen test results with and without ICD. The Heska Feline Heartworm Antibody test revealed an overall prevalence of 3.5% (76/2165), with DiroCHEK® detecting antigen with and without acid treatment in 0.27% (6/2165) of the samples. Samples obtained from cats from the West region of the USA exhibited the highest antibody prevalence, with statistical significance (*P* = 0.044). These results contrast with the CAPC available data between 2017 and 2022, reporting a feline antibody prevalence of 0.61%. The high antibody prevalence percentage (3.5%) indicates that presumably healthy owned cats are at risk for infection with *D. immitis*. Additionally, the number of infected cats may be widely underestimated in non-endemic states due to the perceived lack of risk and thus lack of testing in those regions. Owners and practitioners should increase their awareness of HW in cats, and appropriate use of broad-spectrum veterinary approved parasiticides should be used more frequently in feline patients. While the acid treatment for ICD and antigen detection in this study were inconsistent, more testing with well-characterized feline HW-positive samples should be performed to validate these findings.

### Supplementary Information

**Additional file 1: Table S1.** *Dirofilaria immitis *antibody-positive samples in cats identified in this study.

## Data Availability

The datasets generated during the current study are available from the corresponding author on reasonable request.

## Abbreviations

CAPC: Companion animal parasite council
FAVN: Fluorescent antibody virus neutralization
HARD: Heartworm-associated respiratory disease
HW: Heartworm
HWD: Heartworm disease
ICD: Immune complex dissociation
RFFIT: Rapid fluorescent foci inhibition test
TCA: Trichloroacetic acid
