# Supplementary material for: A nationwide serological survey for Dirofilaria immitis in companion cats in the United States of America: 3.5% antibody and 0.3% antigen positivity
Source: Parasit Vectors. 2023 Aug 24;16:296. doi: 10.1186/s13071-023-05829-7 (PMC10463928; doi:10.1186/s13071-023-05829-7)
Supplement: Supplementary file 1 — Additional file 1: Table S1. Dirofilaria immitis antibody-positive samples in cats identified in this study. [file 13071_2023_5829_MOESM1_ESM.docx]

**Additional file 1: Table S1. *Dirofilaria immitis* antibody positive samples in cats identified in this study**

| **Date of sample submission** | **Age (year)** | **Sex** | **Breed** | **State** |
| --- | --- | --- | --- | --- |
| 08/22/2017 | 6 | Male | Sphynx | NV |
| 10/16/2018 | 6 | Female | Domestic Short Hair | OH |
| 12/06/2018 | 6 | Female | Domestic Short Hair | OH |
| 12/12/2018 | 19 | Female | Domestic Short Hair | LA |
| 01/11/2019 | 18 | Female | Domestic Short Hair | NC |
| 03/06/2019 | 4 | Female | Domestic Short Hair | MS |
| 03/22/2019 | 1 | Female | Domestic Short Hair | TN |
| 03/26/2019 | 17 | Male | Domestic Long Hair | GA |
| 04/04/2019 | 2 | Male | Domestic Short Hair | FL |
| 05/09/2019 | 2 | Female | Domestic Short Hair | AL |
| 05/24/2019 | 3 | Male | Domestic Short Hair | OR |
| 07/09/2019 | 5 | Male | Domestic Short Hair | GA |
| 08/16/2019 | 2 | Male | Domestic Short Hair | MI |
| 12/31/2019 | 7 | Female | Domestic Short Hair | IL |
| 06/18/2020 | 10 | Male | Domestic Short Hair | HI |
| 06/22/2020 | 4 | Male | Domestic Short Hair | LA |
| 06/30/2020 | 9 | Female | Domestic Medium Hair | TX |
| 07/17/2020 | 1 | Female | Sphynx | WA |
| 08/03/2020 | 11 | Female | Domestic Short Hair | SC |
| 08/04/2020 | 3 | Male | Domestic Short Hair | PA |
| 08/05/2020 | 2 | Male | British Longhair | WA |
| 08/21/2020 | 9 | Male | Domestic Long Hair | FL |
| 09/02/2020 | 10 | Female | Domestic Short Hair | HI |
| 09/10/2020 | 2 | Male | Domestic Short Hair | WI |
| 09/11/2020 | 6 | Female | Ragdoll | NC |
| 09/15/2020 | 2 | Male | Domestic Short Hair | FL |
| 11/16/2020 | 2 | Female | Domestic Short Hair | UT |
| 11/17/2020 | 4 | Male | Domestic Short Hair | IN |
| 01/05/2021 | 3 | Female | Domestic Short Hair | GA |
| 01/20/2021 | 1 | Female | Domestic Long Hair | IL |
| 01/21/2021 | 15 | Male | N/A | PA |
| 02/01/2021 | 7 | Male | Domestic Short Hair | HI |
| 02/19/2021 | 7 | Male | Domestic Short Hair | NY |
| 02/23/2021 | 16 | Female | Domestic Short Hair | AL |
| 03/02/2021 | 7 | Male | Domestic Medium Hair | MA |
| 03/02/2021 | 1 | Male | British Shorthair | NY |
| 03/30/2021 | 2 | Male | Ragdoll | AL |
| 04/02/2021 | 2 | Male | Sphynx | AR |
| 04/14/2021 | 5 | Male | Domestic Long Hair | AL |
| 05/21/2021 | 1 | Female | Domestic Short Hair | TN |
| 06/07/2021 | 1 | Female | Siamese | CA |
| 06/22/2021 | 2 | Male | Sphynx | CA |
| 06/29/2021 | 7 | Male | Scottish Fold | VA |
| 06/29/2021 | 2 | Female | Scottish Fold | NY |
| 07/02/2021 | 5 | Male | Domestic Short Hair | CA |
| 07/02/2021 | 5 | Female | Domestic Short Hair | CA |
| 07/26/2021 | 11 | Male | Domestic Long Hair | HI |
| 08/27/2021 | 5 | Male | Domestic Long Hair | GA |
| 08/31/2021 | 4 | Male | Domestic Short Hair | CA |
| 09/08/2021 | 3 | Male | Domestic Short Hair | CT |
| 09/09/2021 | N/A | Female | N/A | NC |
| 09/22/2021 | 5 | Male | Domestic Short Hair | NY |
| 09/30/2021 | 10 | Male | Domestic Short Hair | FL |
| 10/01/2021 | 4 | Male | Domestic Short Hair | GA |
| 11/02/2021 | 9 | Male | Domestic Short Hair | NY |
| 11/04/2021 | 12 | Male | Persian | CA |
| 11/19/2021 | 3 | Male | Ragdoll | CA |
| 11/26/2021 | 1 | Male | British Shorthair | NY |
| 11/30/2021 | N/A | Female | Calico | NC |
| 12/02/2021 | 1 | Male | Domestic Short Hair | MA |
| 12/03/2021 | 2 | Female | Domestic Short Hair | CA |
| 12/09/2021 | 5 | Female | Tabby | AZ |
| 12/28/2021 | 5 | Female | Domestic Short Hair | OH |
| 01/07/2022 | 4 | Female | Persian | CA |
| 01/31/2022 | 1 | Male | Siamese | NY |
| 02/09/2022 | 1 | Male | Domestic Short Hair | CA |
| 02/22/2022 | 4 | Male | Domestic Short Hair | WA |
| 02/25/2022 | 12 | Female | Domestic Long Hair | CA |
| 02/28/2022 | 3 | Male | British Shorthair | AL |
| 03/16/2022 | 5 | Female | Domestic Short Hair | VA |
| 03/24/2022 | 8 | Male | Domestic Short Hair | AL |
| 04/18/2022 | 3 | Female | Domestic Short Hair | GA |
| 04/20/2022 | 2 | Female | N/A | NC |
| 04/22/2022 | 7 | Male | Siamese Mix | CA |
| 04/24/2022 | 1 | Male | Domestic Short Hair | VA |
| 04/26/2022 | 3 | Male | Domestic Short Hair | IN |

* N/A indicates the age or gender of the cats was not available
